# In silico identification of natural products from Traditional Chinese Medicine for cancer immunotherapy

**DOI:** 10.1038/s41598-021-82857-2

**Published:** 2021-02-08

**Authors:** Chuipu Cai, Qihui Wu, Honghai Hong, Liying He, Zhihong Liu, Yong Gu, Shijie Zhang, Qi Wang, Xiude Fan, Jiansong Fang

**Affiliations:** 1 Department of Computer Science, Key Laboratory of Intelligent Manufacturing Technology of Ministry of Education, Shantou University, Shantou, 515000 China; 2 Science and Technology Innovation Center, Guangzhou University of Chinese Medicine, Guangzhou, 510000 China; 3 Clinical Research Center, Hainan Provincial Hospital of Traditional Chinese Medicine, Haikou, 570100 China; 4 Department of Clinical Laboratory, The Third Affiliated Hospital of Guangzhou Medical University, Guangzhou, China; 5 Guangdong Institute of Microbiology, Guangdong Academy of Sciences, Guangzhou, 510000 China; 6 Lerner Research Institute, Cleveland Clinic, Cleveland, OH 44195 USA

**Keywords:** Virtual drug screening, Drug discovery, Natural products, Networks and systems biology

## Abstract

Advances in immunotherapy have revolutionized treatments in many types of cancer. Traditional Chinese Medicine (TCM), which has a long history of clinical adjuvant application against cancer, is emerging as an important medical resource for developing innovative cancer treatments, including immunotherapy. In this study, we developed a quantitative and systems pharmacology-based framework to identify TCM-derived natural products for cancer immunotherapy. Specifically, we integrated 381 cancer immune response-related genes and a compound-target interaction network connecting 3273 proteins and 766 natural products from 66 cancer-related herbs based on literature-mining. Via systems pharmacology-based prediction, we uncovered 182 TCM-derived natural products having potential anti-tumor immune responses effect. Importantly, 32 of the 49 most promising natural products (success rate = 65.31%) are validated by multiple evidence, including published experimental data from clinical studies, in vitro and *in vivo* assays. We further identified the mechanism-of-action of TCM in cancer immunotherapy using network-based functional enrichment analysis. We showcased that three typical natural products (baicalin, wogonin, and oroxylin A) in *Huangqin* (*Scutellaria baicalensis Georgi*) potentially overcome resistance of known oncology agents by regulating tumor immunosuppressive microenvironments. In summary, this study offers a novel and effective systems pharmacology infrastructure for potential cancer immunotherapeutic development by exploiting the medical wealth of natural products in TCM.

## Introduction

Cancer, as the leading cause of death worldwide, is a serious public health issue^1^. In the past several years, cancer immunotherapy has become a remarkable strategy for cancer treatment and received growing attention worldwide in many cancer types. One of the world-renowned examples is the programmed cell death-1 (PD-1)/PD-1-ligand (PDL-1) pathway, which was recognized with the 2018 Nobel Prize in Physiology or Medicine^2^. The number of active agents in the global immuno-oncology pipeline increased by 91% in the last two years (September 2017 to August 2019)^3^. Cancer immunotherapeutic agents with regulating immune evasion (e.g., co-inhibitory checkpoints) or directly stimulating immunogenic pathways (e.g., agonists of costimulatory receptors) have been demonstrated striking clinical responses in patients with multiple types of advanced cancers^4^. Unfortunately, the response rates of current immunotherapy are still modest that only around 20% of cancer patients are responsive^5^. Therefore, more efforts are clearly required to develop novel agents with new mechanism-of-actions (MOAs) for cancer immunotherapy.

Traditional Chinese Medicine (TCM) has a long history of clinical adjuvant application for cancer treatment^6^. Accumulating clinical evidence and pharmacological studies have demonstrated the great potential of Chinese herbs as well as their extracts as novel and effective cancer interventions^7^. A number of natural products derived from TCM, such as berberine^8,9^, baicalin^10^, and resveratrol^11^, have been reported to have regulatory effects on tumor microenvironments, including regulation of macrophage activation, cytokine secretion, and T cell differentiation^12^. Natural products with abundant chemical scaffolds and promiscuous target profiles, are emerging as an invaluable chemical library for discovering bioactive cancer immunotherapeutic candidates^13–15^.

Due to the heterogeneous ingredients and multiple target interactions, conventional experimental assays are labor-consuming and time-costing^16^. Quantitative and systems pharmacology, as a multidisciplinary strategy for the emerging development of efficacious drugs via integration of experimental assays and computational approaches^17^, has been widely applied to identify active natural products and elucidate their underlying mechanisms against complex diseases^18^, such as aging-associated disorders^19,20^, coronary artery diseases^21^, and cancers^16,22^. For example, Huang et al. had successfully uncovered wogonoside as an angiogenesis inhibitor in treating triple-negative breast cancer (TNBC) using systems pharmacology approach and validated it by in vitro and *in vivo* assays^23^. Cheng et al. predicted an integrative, network-based systems pharmacology framework for personalized drug repurposing^22^. They successfully identified ouabain, a FDA-approved plant-derived toxic substance in treatment of non-small cell lung cancer^22^. Altogether, quantitative and systems pharmacology approaches offer effective strategies for discovering potential natural products for the development of cancer therapies, including immunotherapy.

**Figure 1:**
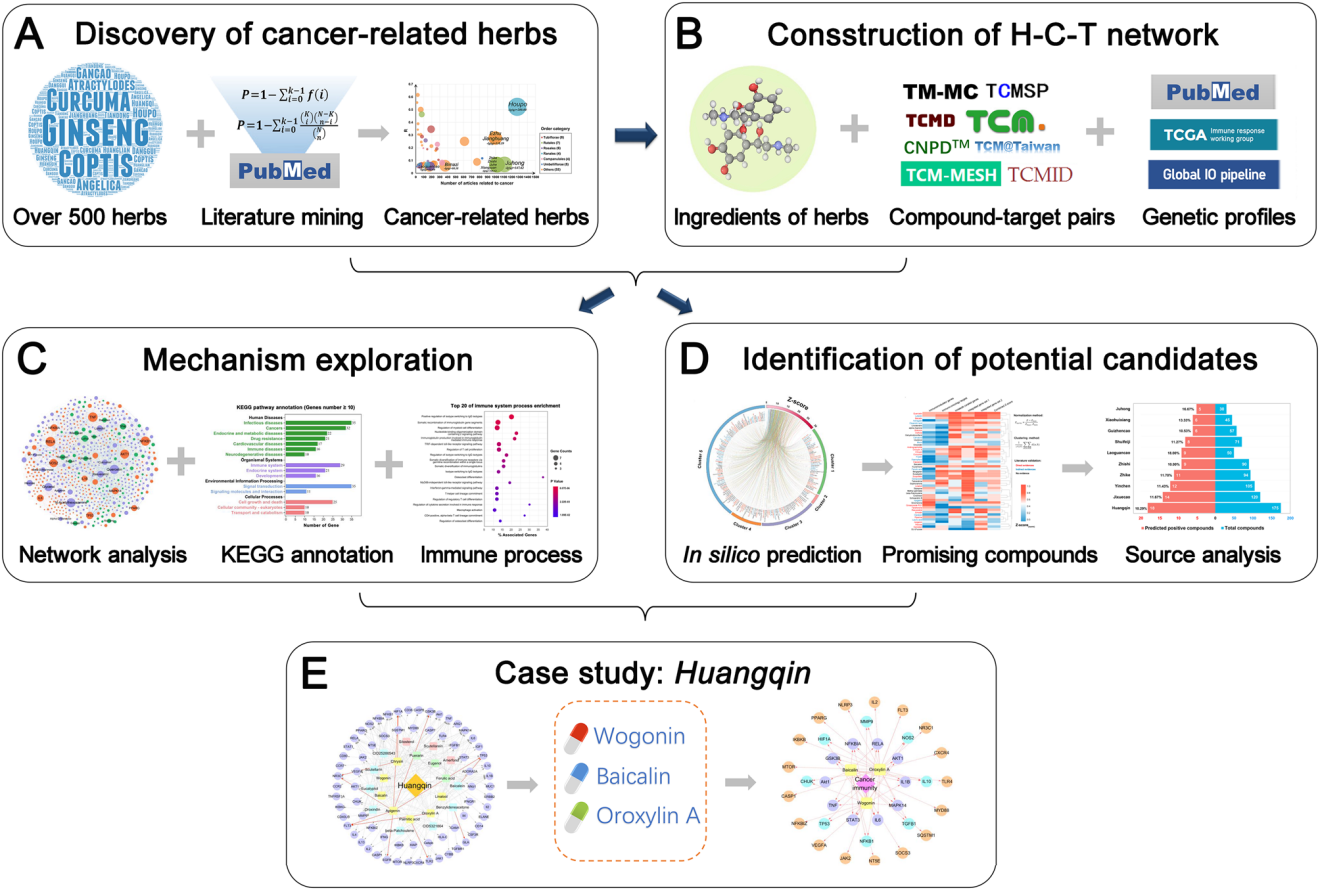
Schematic diagram of an integrated systems pharmacology framework for identification of potential cancer immunotherapeutic natural products from Traditional Chinese Medicine (TCM). (**A**) Literature mining-based discovery of cancer-related Chinese Herbs from PubMed database. (**B**) Construction of cancer immune response-related herb-compound-target (H-C-T) network of TCM- derived natural products. (**C**) Mechanism-of-action exploration of TCM in cancer immunotherapy. (**D**) Identification of new cancer immunotherapeutic candidates from natural products. (**E**) *Huangqin *(*Scutellaria baicalensis Georgi*) as a case study to highlight its main active constituents and elucidate the underlying mechanisms of immunotherapeutic against cancer.

In this study, we proposed an integrated systems pharmacology-based framework, as illustrated in Fig. 1, to uncover novel TCM-derived cancer immunotherapeutic agents. Specifically, we firstly identified Chinese herbs highly related to cancer through a large-scale literature mining from PubMed database. Subsequently, a global herb-compound-target (H-C-T) network of TCM derived-natural products was constructed by integrating experimentally reported cancer immune response-related (CIR) genes, herb-compound pairs, and compound-target interactions (CTIs) extracting from our previous consolidated databases^16,23^. On the basis of the H-C-T network, we next systematically inspected the cancer immunotherapeutic mechanisms of TCM, and built in silico models with high accuracy to predict natural products for potential immunotherapy. Overall, this study offers a powerful systems pharmacology approach for identification of promising candidates from natural products for development of tumor immunotherapy.

## Results

### A landscape of Chinese herbs in cancer

**Figure 2:**
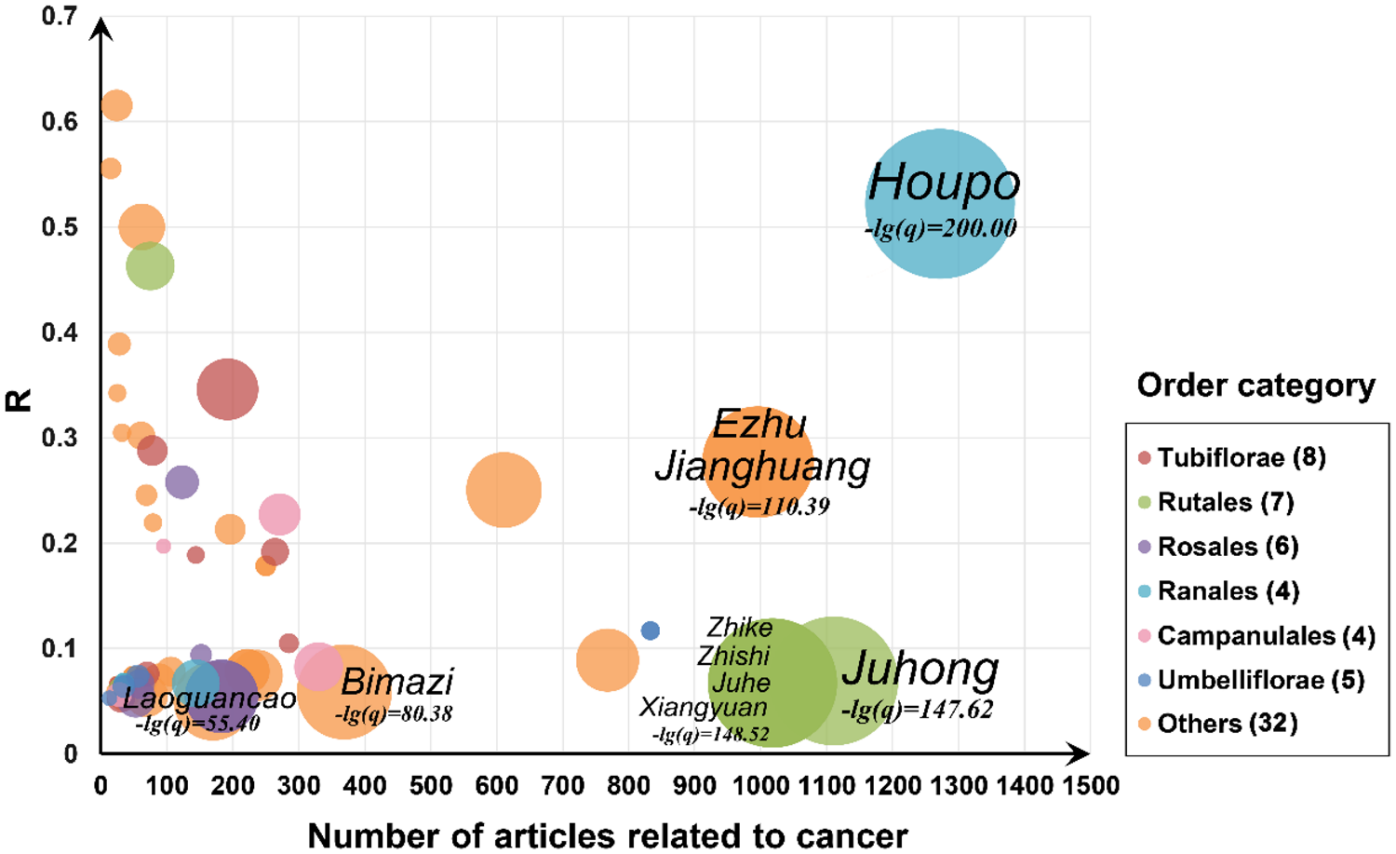
Statistics and association analysis results between 66 herbs and cancer based on literature mining from PubMed database. The herbs are prioritized according to the negative logarithm (base 10) of their adjusted-*P* (*q*) values, and the size of circles denotes the value of *− lg*(*q*). R represents the ratio of (cancer-herb-related articles)/(herb-related articles). Labels of the top 10 herbs with highest *− lg*(*q*) values are displayed. The zero value of *q* was replaced by an extremely small positive value (1E-200) for calculating negative logarithm.

Via large-scale literature mining analysis, we found that 66 of the 525 investigated herbs showed significant associations with cancer (*q* < 0.01, R > 0.05, Supplementary Table S1). As shown in Fig. 2, the top 10 herbs with highest *− lg*(*q*) values are commonly used in the clinical adjuvant treatment of cancer, while their extracts also have been confirmed to possess anti-tumor effect by *in vivo* and in vitro assays^7^. For example, magnolol isolated from the ranking first herb *Houpo* (*Magnolia officinalis*, *− lg*(*q*) = 200.00) had been proved to exert broad anti-cancer activity by reducing proliferation, suppressing differentiation, counteracting metastasis, and restraining angiogenesis^24–27^. Besides, *Jianghuang* (*Curcuma longa*, *− lg*(*q*) = 110.39), a main source of the polyphenolic compound curcumin, is a natural pleiotropic herb used in treatment and prevention of multiple diseases, including cancer^12^. Put together, the literature mining narrows down the scope of investigation for highlighting the most valuable herbs highly related to cancer, which deserves to further identify their potential ingredients as cancer immunotherapeutic agents.

### Herb-compound-target (H-C-T) network analysis of natural products in cancer immunotherapy

**Figure 3:**
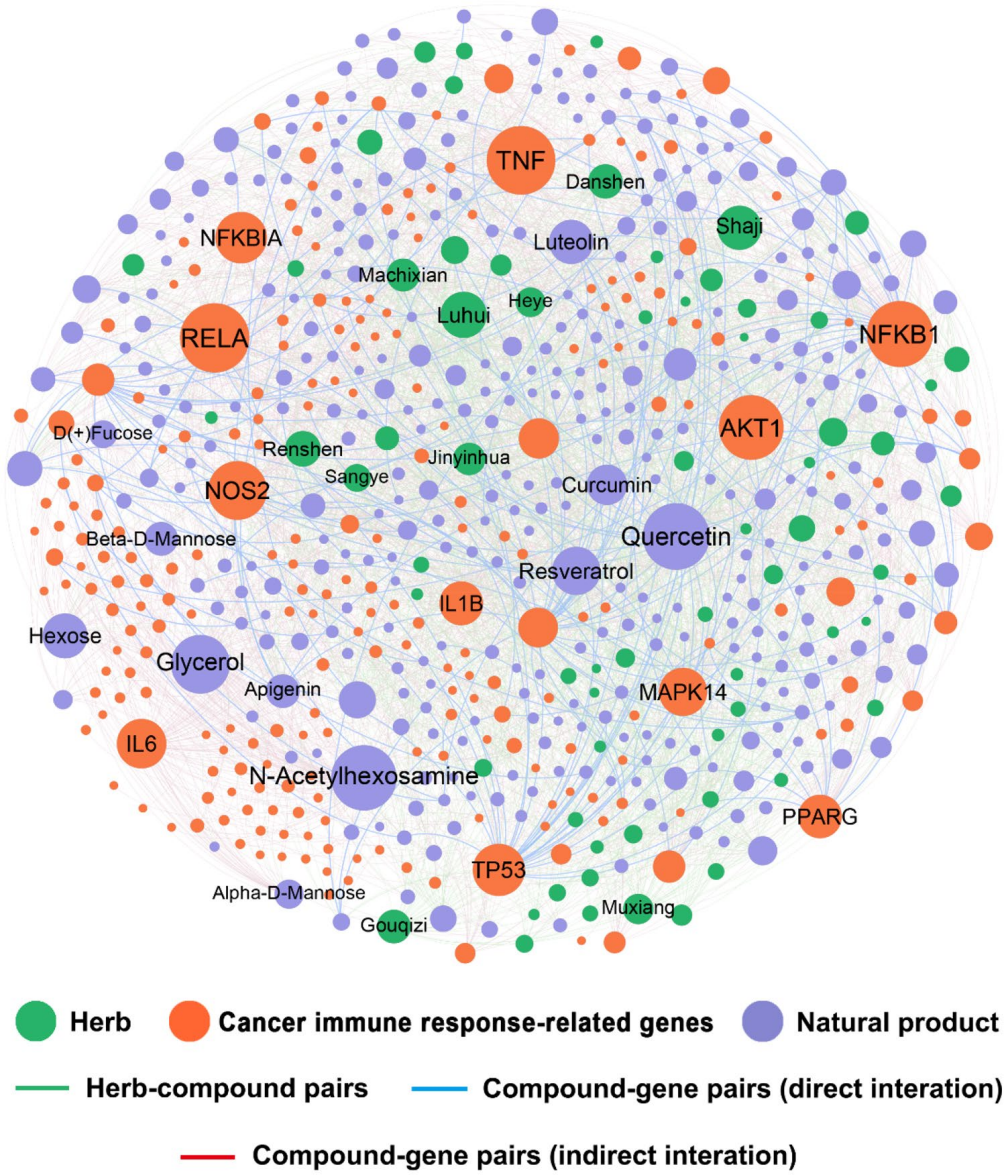
Herb-compound-target (H-C-T) network for natural products derived from 66 cancer-related herbs in cancer immunotherapy. The label font size and node size are proportional to degree (connectivity) of the item. Labels of the top 10 herbs, compounds, and targets with the highest degree are displayed. For the compound-target interactions that were labeled as direct and indirect interactions simultaneously, the direct ones were preserved.

We firstly acquired the constituent compounds and corresponding targets of the 66 cancer-related herbs from the integrated H-C-T database, resulting in 12,562 interactions connecting 766 unique compounds with 3273 protein targets (Supplementary Table S2). Subsequently, an anti-cancer immune response-related H-C-T network was constructed by extracting the compound-protein interactions related to CIR genes. As shown in Fig. 3, the H-C-T network is composed of 648 nodes (66 herbs, 344 compounds, and 238 targets) and 3270 edges (2095 CTIs and 1175 herb-compound pairs). Among the 2095 CTIs, 186 with direct binding affinity are labeled as direct interactions, while the rest 1909 are regarded as indirect interactions. Network analysis shows that these natural products are connected to multiple cancer immunity-related targets with the average degree (*K*) of 6.1 for each natural product, which indicates that they may have important influence on cancer immunotherapy. For instance, quercetin possessing the third largest number of target connections (*K* = 61), has been reported to promote the immune response and repress the growth of murine leukemia WEHI-3 cells^28^In vitro and *in vivo* studies also suggested that resveratrol (*K* = 56) could suppress lung cancer growth through inhibiting M2 polarization of human monocyte derived macrophage. For the 238 targets, the average degree (*D*) in this network is 8.8. Among them, RELA (*D* = 93) exhibited the highest connections to natural products, followed by TNF (*D* = 92) and NFKB1 (*D* = 88). Relevant studies show that these targets play essential roles in tumor immunity^29,30^.

### Mechanism-of action of natural products in cancer immunotherapy

**Figure 4:**
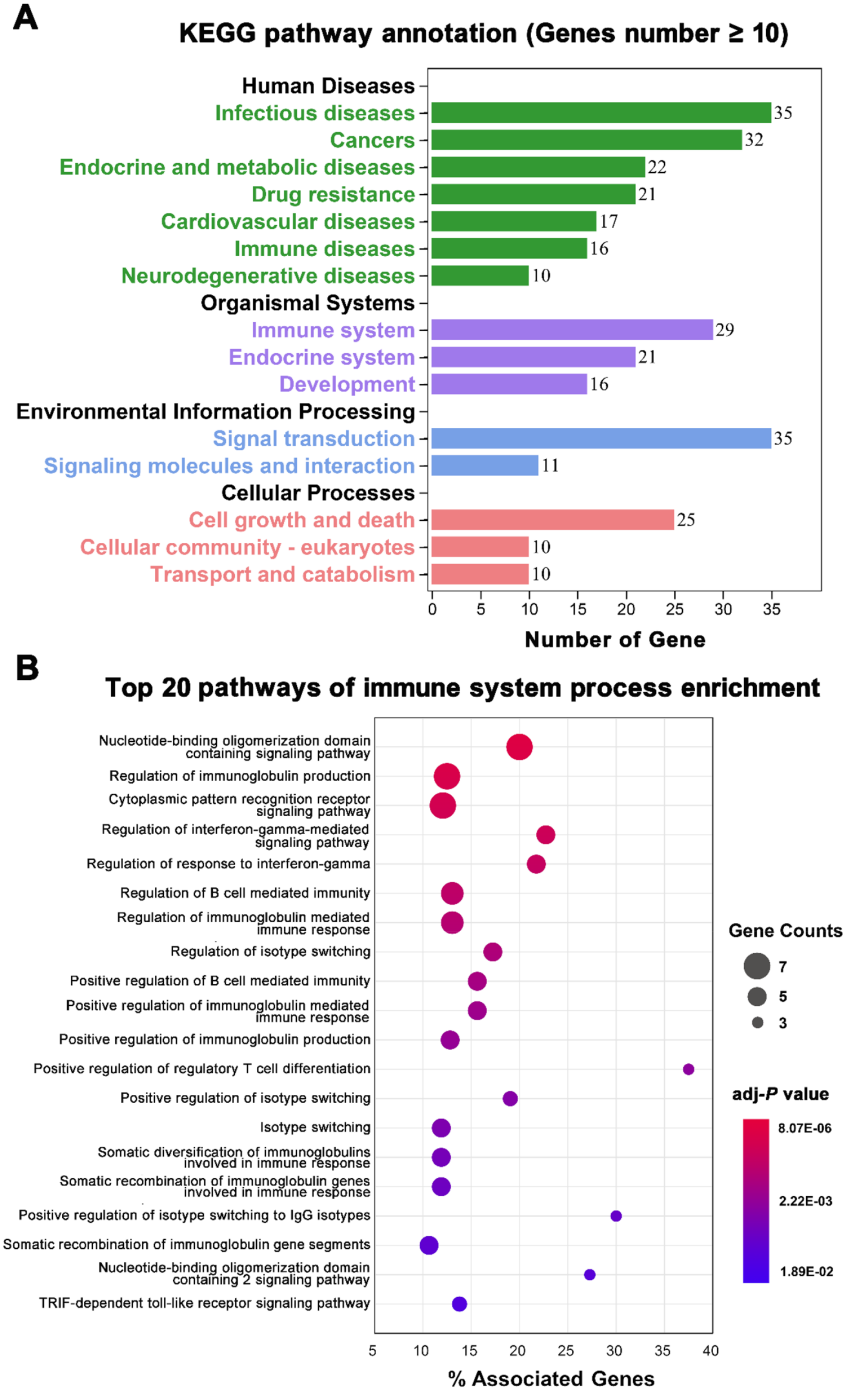
Enrichment analysis of the 41 cancer immune response-related genes with degree higher than or equal to 10 in the herb-compound-target (H-C-T) network. (**A**) Kyoto Encyclopedia of Genes and Genomes (KEGG) pathway annotation. Different KEGG classes are displayed in various colors. (**B**) Immune system process enrichment.

To elucidate the molecular mechanisms underlying the cancer immunotherapeutic effects of TCM, 41 CIR genes with degree greater than or equal to 10 (*D* ≥ 10) in the H-C-T network (Supplementary Table S3) were selected to perform enrichment analysis (Fig. 4). Kyoto Encyclopedia of Genes and Genomes (KEGG) pathway^31^ annotation (Fig. 4A) shows that a large number of targets (T) are involved in cancer (T = 32) and immune system (T = 29) related pathways, indicating their high correlativity with cancer immunotherapy of TCM. According to the enrichment analysis results of environment information processing and cellular process, these CIR genes may participate in the regulation of signal transduction and modulate cell growth and death.

In this study, we mainly aim at the immune-related processes and try to dig out the underlying cancer immunotherapeutic mechanisms of TCM. Thus, the immune system process enrichment analysis was further performed using ClueGO^32^. We obtained 31 significant immune system pathways with adjusted *P*-values (*q*) less than 0.05 (corrected with Bonferroni step down) as well as associated genes proportion higher than 10% (Supplementary Table S3). Figure 4B presents the top 20 enriched immune system pathways. Most of these pathways were associated with cancer immunotherapy, which can activate immune responses, including positively regulation of T cell differentiation (*q* = 6.03 × 10^–3^), B cell-mediated immunity (*q* = 2.22 × 10^–3^), immunoglobulin production (*q* = 5.38 × 10^–3^), and secretion of other cytokines (*q* = 0.0499). Besides, tumor immune escape is also an important point. Enrichment analysis demonstrated that these anti-cancer herbs may prevent from tumor immune evasion through regulating immune system processes such as TRIF-dependent toll-like receptor signaling pathway (*q* = 0.0189) and interferon-gamma-mediated signaling pathway (*q* = 3.48 × 10^–4^). Detailed immune system process enrichment analysis results of the 41 cancer CIR genes are provided in Supplementary Table S3. In general, TCM-derived natural products may have multi-faceted impact on cancer immunotherapy both via “immune enhancement” and “immune normalization” strategies^4^. Thus, we next turned to identify novel cancer immunotherapeutic agents from TCM-derived natural products.

### Systems pharmacology-based prediction of potential natural products for cancer immunotherapy

Through integrating the compound-target network of 66 cancer-related herbs and five sets of CIR gene sets, we further built five statistical network models for discovering novel cancer immunotherapeutic agents from natural products. By applying the threshold of adjusted *P*-value (*q*) < 0.01, the five in vitro models based on different gene sets computationally identified 71–155 natural products as promising candidates, respectively (Supplementary Table S4).

For evaluating the predictive accuracy of the in vitro models, we systematically retrieved previous literature evidence from PubMed for the predicted positive natural products in cancer immunotherapy (Supplementary Table S5). Here, the direct evidence refers to a natural product exerting anti-cancer immune responses, while the indirect evidence denotes a natural product enhancing immune response and possessing anti-cancer potential by *in vivo* assays simultaneously. As listed in Supplementary Table S4, all the five models achieve high predictive accuracy with the success rates of over 50%, suggesting the reliability of computational models. The natural products without experimental verification, especially those have indirect evidence, offer new potential immunotherapeutic candidates that deserve to be further experimentally validated.

**Figure 5:**
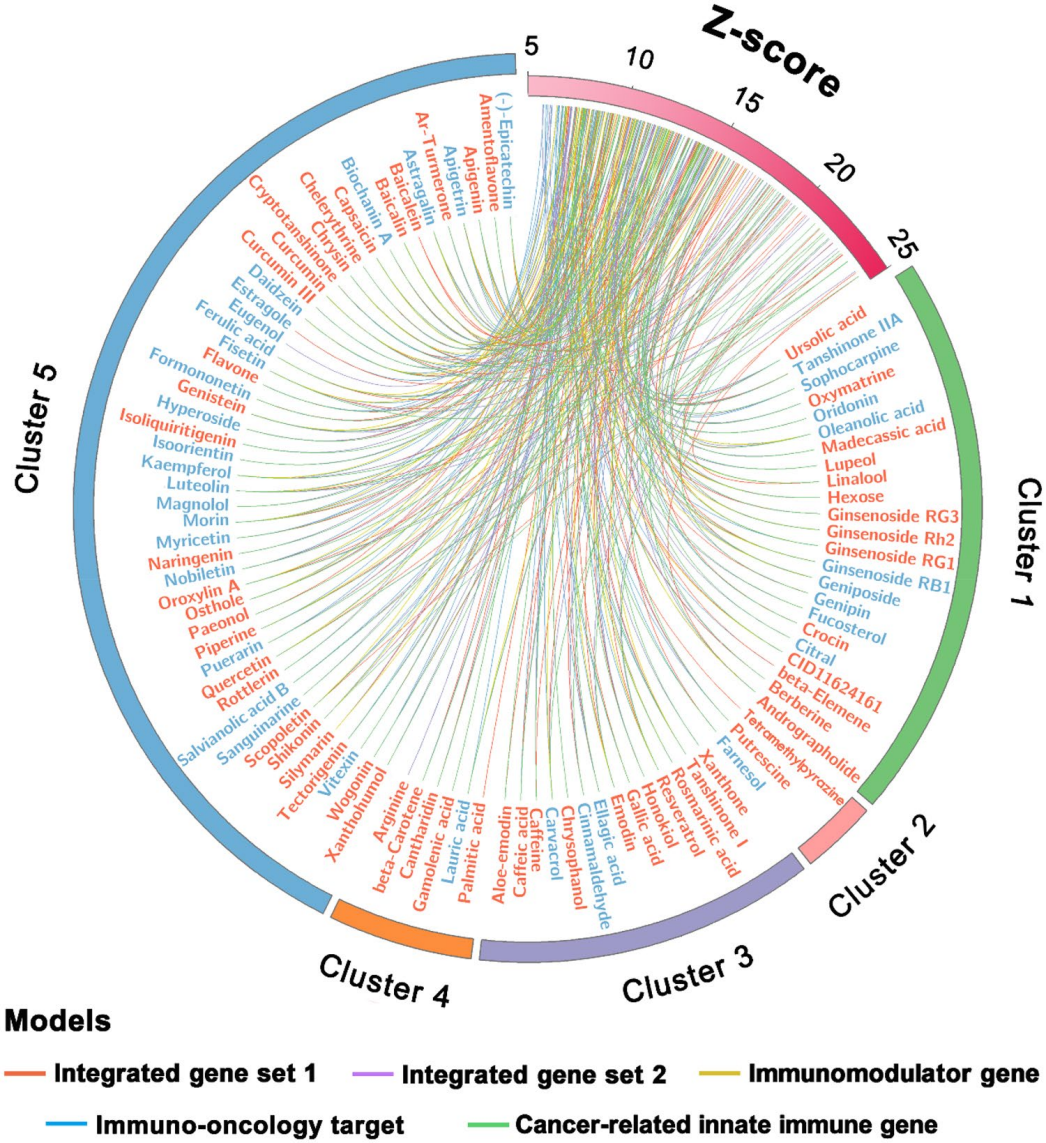
Circos plot exhibiting the 95 predicted natural products (adjusted-*P* < 0.01) with direct evidence (in red font) or indirect evidence (in blue font). Natural products are grouped by chemical scaffold clustering analysis (Supplementary Fig. S1). The predicted associations from different statistical network models are connected lines in various colors. The full version exhibiting all the 182 predicted natural products (adjusted-*P* < 0.01) is provided in Supplementary Fig. S2. The circos plots were drawn using Circos (v0.69)^37^.

**Figure 6:**
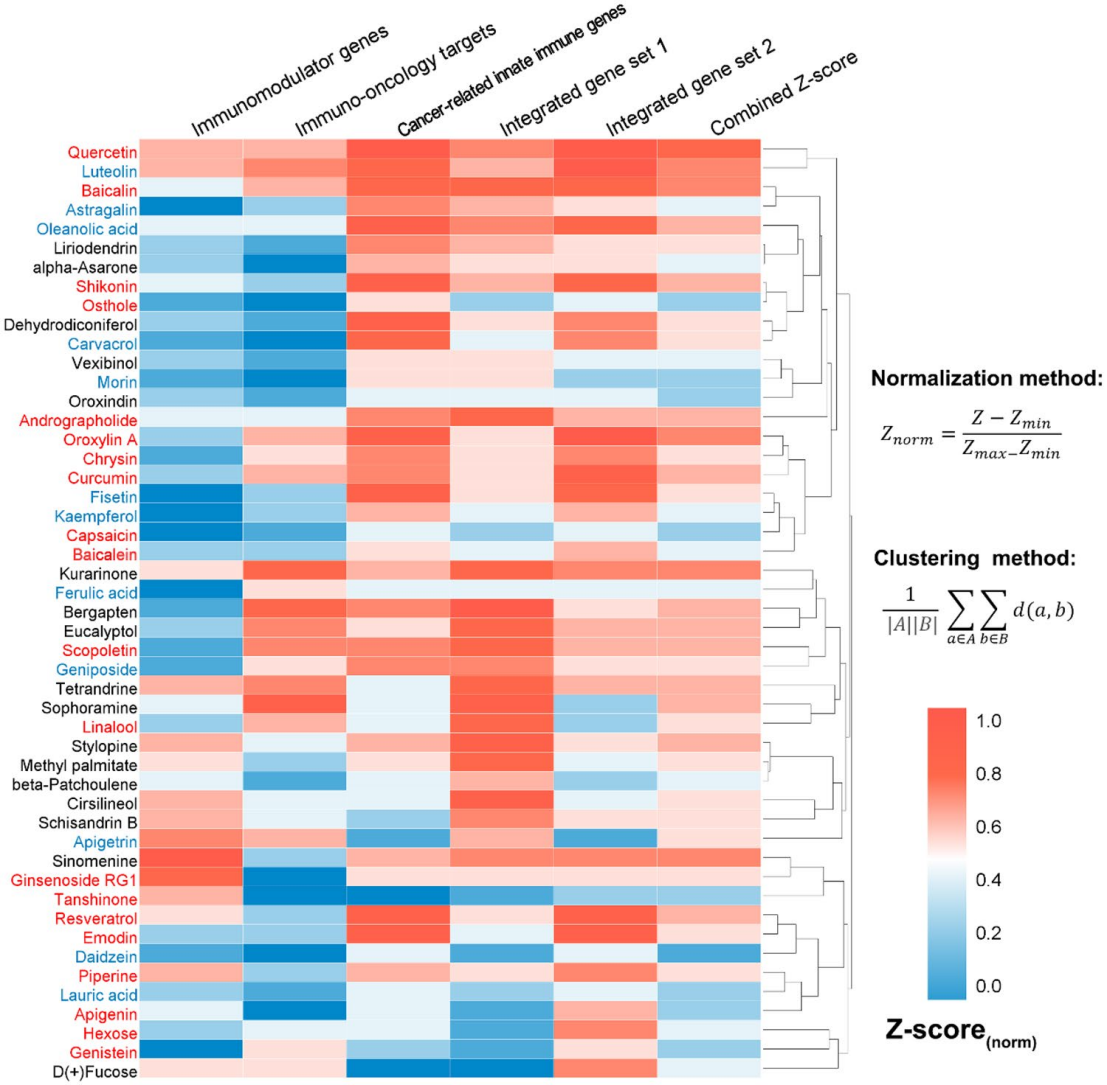
Heatmap highlighting the 49 natural products simultaneously predicted as potential cancer immunotherapeutic candidates by all the five statistic network models. The relative metric combined Z-score was calculated for each natural product by averaging the normalized Z-scores predicted by the five models. Natural products validated by direct evidence and indirect evidence are highlighted in red and blue font, respectively. The heatmap was drawn using HemI (v1.0)^38^.

Due to the similar and satisfactory performance of the five models, we merged their prediction results for further discussion. In total, we obtained 182 natural products for further analysis. Clustering analysis was performed to cluster their chemical scaffolds into 5 groups by calculating the root-mean-square value of the Tanimoto distance based on FCFP_6 fingerprint (Supplementary Fig. S1A). The structures of the five cluster centers, including ginsenoside Rh2, 1-Tetradecanol, chrysophanol, palmitic acid, and apigenin are given in Supplementary Fig. S1B. As presented in Fig. 5 and Supplementary Fig. S2, among the 182 predicted natural products, 59 were validated by direct evidence, while 36 were indicated by indirect evidence, with an overall success rate of 52.20% (95/182). Most of the natural products were identified as potential cancer immunotherapeutic agents by more than one statistical network models. We next turned to focus on the 49 natural products that had been simultaneously predicted to be positive by all the five models (Fig. 6). Evidence from literature demonstrates that the success rate is increased to 65.31% (32/49), with 20 and 12 of the 49 natural products possess direct evidence and indirect evidence, respectively. We calculated the combined Z-score to provide a relative metric for comparison by combining the predictions of the five models (see the details in Fig. 6). The top 10 natural products prioritized by combined Z-score are quercetin, luteolin, baicalin, kurarinone, sinomenine, oroxylin A, bergapten, oleanolic acid, tetrandrine, and resveratrol (Supplementary Table S6). A growing body of experimental evidence has indicated their potential anti-cancer effects through cancer immunity such as luteolin^33–36^. Put together, the proposed systems pharmacology-based approach provides promising cancer immunotherapeutic candidates from natural products which deserve further preclinical validation.

### Case study: mechanism-of-action of anti-tumor immunotherapy by *Huangqin* (*Scutellaria baicalensis georgi*)

We systematically analyzed the source of the 182 predicted positive natural products (Supplementary Fig. S3A). We found that there were 16 herbs possessing a positive ingredients proportion higher than 10% (Supplementary Table S7). Among them, *Huangqin *(*Scutellaria baicalensis*) has the highest number (*n* = 18) of potential cancer immunotherapy ingredients (Supplementary Fig. S3B). We thus selected *Huangqin* as a case study to showcase its anti-tumor immunotherapeutic mechanisms.

**Figure 7:**
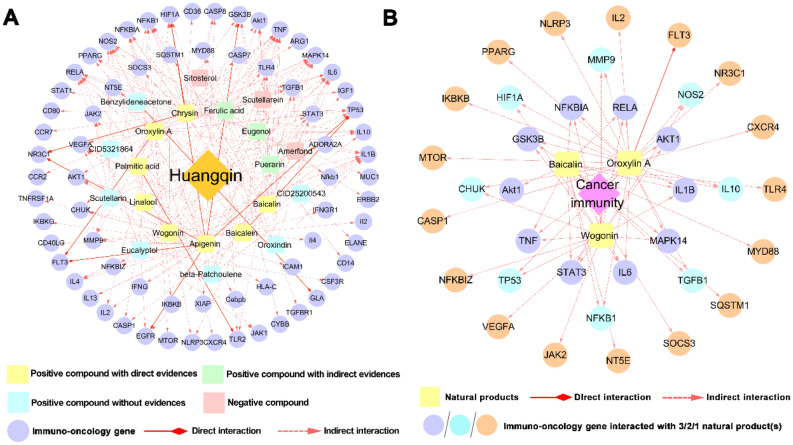
Compound-target (C-T) network of *Huangqin* (*Scutellaria baicalensis Georgi*) in cancer immunotherapy. (**A**) C-T network for 204 interactions connecting 21 compounds of *Huangqin* and 66 cancer immune response-related (CIR) genes. (**B**) C-T network for 60 compound-target interactions connecting three main bioactive flavones of *Huangqin* (baicalin, wogonin and oroxylin A) and 34 CIR genes.

*Huangqin* and its monomer extracts had been widely validated by clinical trials and experiments for the treatment of various cancers^39^. For example, Skullcapflavone I, a flavone compound extracted from *Huangqin*, has been demonstrated to exert anti-cancer effect on lung cancer cells through inactivating PI3K/AKT/mTOR signaling pathway^40^. Although massive anti-tumor studies of *Huangqin* have emerged^39^, there are few researches focusing on its immunotherapeutic properties. Thus, in this part, we aim to systematically elucidate the anti-cancer mechanism of *Huangqin* and highlight its main active constituents in cancer immunotherapy (Fig. 7).

As illustrated in Fig. 7A, the cancer immunity compound-target network of *Huangqin* consists of 204 interactions connecting 21 natural products and 66 CIR genes. Among the 21 natural products, 18 out of them were predicted as positive, suggesting their high correlation with cancer immunity. Eight of them were validated by direct literature evidence, while three were supported by indirect evidence. For instance, linalool has been reported to exhibit cytotoxic effects by activating anti-tumor immunity^41^. Among the eight predicted natural products with direct evidence, baicalin, wogonin, and oroxylin A are the main bioactive flavones of *Huangqin* detected by ultra-high-performance liquid chromatography (UHPLC) method^42^. *In vivo* studies of *Huangqin* against multiple tumors also revealed that predominantly conjugates and aglycones of them could be found in plasma^39^, suggesting their important role in oncotherapy. Therefore, we selected these three typical natural products (baicalin, wogonin, and oroxylin A) to develop a specific anti-cancer immune response-related compound-target network for further investigating the underlying cancer immunotherapeutic mechanism of *Huangqin*.

As displayed in Fig. 7B, the specific network of the 3 natural products is composed of 60 CTIs. Interestingly, we found that baicalin, wogonin, and oroxylin A shared multiple overlapped CIR genes, including RELA, IL1B, MAPK14, IL6, STAT3, TNF, GSK3B, and NFKBIA, which indicates that *Huangqin* may exert cancer immunotherapeutic effect through the synergistic action of these components. These overlapped targets had been validated to play significant roles in the therapeutic action of *Huangqin* against many types of cancer^43–45^. Overall, the systems pharmacology approach applied here uncovers the main active constituents and key protein targets of *Huangqin* in cancer immunotherapy.

## Discussion

Cancer immunotherapy, which was proven to elicit clinically meaningful and durable responses in multiple tumor types, has led to a new era of cancer treatment. In this study, we proposed a novel integrated quantitative and systems pharmacology infrastructure to identify potential agents of natural products for cancer immunotherapy and decipher the underlying molecular mechanisms. This work explores the following new cancer immunotherapy characteristics of TCM: (i) We identified 66 significant cancer-related herbs through a large-scale literature mining of PubMed database; (ii) A cancer immune response-related H-C-T network was constructed by integrating experimentally reported targets and multiple CIR gene sets. MOAs of various immunotherapeutic effects of TCM-derived natural products are investigated through current network; (iii) in vitro models yielding high accuracy are developed to prioritize novel anti-cancer natural products in cancer immunotherapy.

Network analysis showcased the great potential of TCM-derived natural products in immuno-oncology and highlighted the common targets related to tumor immunoregulation. Among the 238 targets in H-C-T network, RELA (*D* = 93) and TNF-α (*D* = 92) owned the largest connections to natural products. Indeed, emerging evidence has suggested their anti-tumor immunoregulation effect. For example, RELA can drive tumor-associated macrophages to suppress CD8+ cytotoxic T lymphocytes for tumor promotion, indicating a RELA-targeted immunotherapy for lung cancer^29^. Besides, TNF-α, as the prototype of the TNF superfamily cytokines, has been validated to enhance the differentiation of Th9 cell and exert anti-tumor immunity effect via TNFR2-dependent pathways^30^. Moreover, the in vitro models have identified 49 novel anti-cancer natural products that are highly related to cancer immunotherapy. For example, luteolin, a flavonoid ranking second among the 49 predicted natural products (Fig. 6), has not yet been reported for its cancer immunotherapeutic action. Previous *in vivo* study confirmed that luteolin exerted anti-cancer effects in multiple cancer types^33–35^. In addition, a recent study showed that luteolin enhanced immune cell functions, including proliferation, cytotoxic T lymphocyte (CTL) activity and natural killer (NK) cell activity in isolated murine splenocytes^36^, indicating its potential in cancer immunotherapy.

Based on the global H-C-T network and the in vitro predictive models, current quantitative and systems pharmacology framework can also be applied to extend the cancer immunotherapy knowledge of single herb. We highlighted three main ingredients of *Huangqin* (baicalin, wogonin, and oroxylin A) that might be able to regulate tumor immunosuppressive microenvironments. Subnetwork analysis indicated their overlapped CIR genes may exert synergistic action against multiple cancer. Taking STAT3 as an example, experimental research had demonstrated that both baicalin and wogonin could inhibit tumor growth via acting on STAT3^43,44^. A recent study revealed that baicalin decreased STAT3 activity and further downregulated IFN-γ-induced PD-L1 expression to promote anti-tumor immunity^43^. Furthermore, wogonin was also reported to inhibit tumor growth and enhance immune system activation by suppressing STAT3 signaling^44^. In addition, previous study showed that oroxylin A suppressed the generation of Tregs in lung cancer environment through modulating NF-κB signaling^45^. These experimental results support our in vitro findings to some extent.

Several limitations of this work should be recognized. First, although we have integrated a wide range of compound-protein interactions from published literature and publicly available databases, the potential incompleteness of current networks may be inevitable. Recent studies show that the importing of computationally predicted interactions inferred by balanced substructure–drug-target network-based inference may help to improve the performance of current in vitro models^14,46^. Second, current approach can only predict the potential immunotherapeutic effect of natural products targeting known CIR gene-encoded proteins. Integrating systems biology resources can facilitate the identification of the growing potential CIR protein targets by indirectly locating their neighbours in the human protein–protein interaction network, gene regulatory network, or biological pathways^19^. Third, a gap may exist between the activity of a specific molecule observed in a model system and the true effect in a living organism. For example, the absorption, distribution, metabolism and excretion (ADME) properties that reflect the pharmacokinetics and disposition of chemicals, are critical related to the real efficacy of chemicals *in vivo* situation^47^. Hence, a tougher standard that integrated multi-dimensional analysis, such as ADME prediction, drug-likeness assessment, or pharmacokinetics and pharmacodynamics (PK/PD) analysis, should be applied for further narrowing the gap. Finally, although recent experimental study had validated some of the predicted positive natural products preliminarily, further *in vivo* experiments are necessary to validate the predicted cancer immunotherapeutic effects and molecular mechanism of natural products before advancement to translational studies or clinical trials.

## Conclusion

In summary, our systems pharmacology-based approaches proposed in this study show promise in the in vitro identification of potential anti-cancer natural products acting on immune microenvironment. This network-based approach has provided useful methodology for lead identification purposes of tumor immunotherapy. In combination with in-depth experimental validation, we believe that this framework could serve as a valuable and complementary workflow to shorten the time and improve efficiency for identifying promising anti-tumor immunotherapy candidates, and could be applied in other complex diseases.

## Materials and methods

### Large-scale literature mining of cancer-related Chinese herbs

We performed a comprehensive literature mining of PubMed database to extract Chinese medicinal herbs that highly correlated with cancer. We initially collected 525 clinically used herbs included in *Pharmacopoeia of the People's Republic of China 2015* and annotated their English scientific terms according to the *Flora Reipublicae Popluaris Sinicae (FRPS)*. To balance the bias of different literature number on various herbs, a variable R was imported to evaluate the correlation between herbs and cancer by calculating the ratio of (cancer-herb-related articles)/(herb-related articles). We further applied *P*-value to statistically analyze the chance probability of co-occurrences of each herb and cancer in at least k papers, as Eq. (1) described:

1 $$P = 1 - \mathop \sum \limits_{i = 0}^{k - 1} f\left( i \right) = 1 - \mathop \sum \limits_{i = 0}^{k - 1} \frac{{\left( {\begin{array}{*{20}c} K \\ i \\ \end{array} } \right)\left( {\begin{array}{*{20}c} {N - K} \\ {n - i} \\ \end{array} } \right)}}{{\left( {\begin{array}{*{20}c} N \\ n \\ \end{array} } \right)}} ,$$

where *N* is the total number of papers in PubMed (*N* = 29.0 million, accessed in Jun 10, 2019), *K* stands for the number of literature related to cancer (*K* = 3,874,763), *n* and *k* represent the amounts of papers for a specific herb and its corresponding studies on cancer, respectively. Subsequently, the nominal *P*-values were corrected as adjusted *P*-values (*q*) using R based on the Benjamini–Hochberg approach^48^. In this study, herbs with *q* value lower than 0.01 as well as R values greater than 0.05 are considered significantly correlated with cancer.

### Manual curation and integration of multiple cancer immune response-related (CIR) gene sets

We collected CIR gene sets from multiple resources: (i) Immuno-oncology targets (IOs) in the current global IO pipeline^49^. We removed targets that possess less than 3 agents, resulting in 138 IO targets; (ii) Cancer immunomodulators (IMs) gene set obtained from a literature review, which consists of 78 genes that had been reviewed and confirmed by tumor immunologists from The Cancer Genome Atlas (TCGA) immune response working group^50^. Since recent accumulating evidence have showed innate immune genes play crucial roles in cancer immunology^51,52^, we further curated: (iii) cancer-related innate immune gene set (INs) containing 226 genes identified by various experimental assays (Supplementary Table S8). All the genes/targets were converted into unified Gene Entrez ID and duplicated data in each gene set were eliminated. Fisher’s test method showed that there are significant correlations (*p* < 10^–5^) among the three gene sets at a biological level (Supplementary Fig. S4).

Furthermore, we integrated two additional gene sets based on current three CIR gene sets. The first integrated gene set (IG-1) contains 57 genes that are included in at least 2 out of 3 CIR gene sets, while the second integrated gene set (IG-2) containing 381 genes is the union set of all the three CIR gene sets.

### Construction of herb-compound-target (H-C-T) networks for natural products

We built a comprehensive herb-compound network database by integrating data from multiple TCM databases^21^. The constituent compounds of each Chinese herb were manually extracted from six publicly available TCM data sources, including Traditional Chinese Medicine integrated database (TCMID)^53^, Traditional Chinese Medicine Systems Pharmacology (TCMSP)^54^, Traditional Chinese Medicine database (TCMDb)^55^, Traditional Chinese Medicine database@Taiwan (TCM@Taiwan)^56^, TM-MC^57^, and TCM-MESH^58^. All compounds were transformed to canonical SMILES format and compounds with identical structures were merged.

We further constructed a H-C-T database of natural products through combining physical binding (direct) and functional (indirect) targets of natural products from multiple data sources. The physical binding targets were collected from ChEMBL (v21)^59^ and BindingDB (accessed in September 2017) (accessed in September 2017) databases, while functional targets were obtained from literature and three databases: STITCH 5 (accessed in September 2017)^60^, TCMID 2.0^61^, and Herbal Ingredients’ Targets Database (HIT)^62^. In total, more than 2,000 natural product-specific pharmacological articles (dating from January 2009 to December 2017)^23^ were evaluated. For the direct targets, only interactions with binding affinity data [inhibitory constant (Ki), dissociation constant (Kd), half maximal inhibitory concentration (IC_50_) or half maximal effective concentration (EC_50_)] lower than 10 μm were retained^16^. After removing targets without standard UniProt accession number, 38,220 unique interactions connecting 3882 natural products and 5643 human proteins were finally obtained. The detailed descriptions about the CTIs curation are provided in our previous works^16,23^.

### Network-based statistical models

In this study, we aimed to identify potential immunotherapeutic natural products from cancer-related TCM herbs. Five integrated statistical models were built independently through incorporating compound-target (C-T) networks into the five sets of curated CIR gene sets. It is plausible to hypothesize that a natural product exhibits a high possibility to possess cancer immunotherapeutic effect if its targets are more likely to be CIR gene-encoded proteins. The null hypothesis asserts that the targets of a natural product randomly located at CIR-encoded proteins across the human proteome. We firstly eliminated natural products without any CIR-encoded protein according to the five gene sets. Ions and organic solvents were also removed. Subsequently, permutation test as Eq. (2) given was performed to calculate the statistical significance of a natural product to be prioritized for potential association with cancer immunotherapy:

2 $$P = \frac{{\# \{ Sm\left( p \right) > Sm\} }}{{\# \left\{ {total\,permutations} \right\}}} .$$

A nominal *P* was computed for each natural product by counting the amount of the permutations (*Sm (p)*) larger than observed CIR genes (*Sm*). Here we randomly extracted *x* genes (*x* refers to the number of genes in each CIR gene set) from protein products at the genome-wide scale that includes 20,462 human protein-coding genes from the National Center for Biotechnology Information database^63^ for 100,000 times. Natural products with adjusted *P*-values (*q*, corrected by Benjamini–Hochberg approach)^48^ lower than 0.01 were regarded as significantly related to cancer immunotherapy. Besides, Z-score was further calculated by Eq. (3) to quantify the potential association of each natural product and cancer immunotherapy during permutation testing:

3 $$Z = \frac{x - \mu }{\sigma },$$

where *x* is the actual number of observed CIR genes targeted by a specific natural product, *μ* is the average number of observed CIR genes targeted by a specific natural product during 100,000 permutations, and σ is the standard deviation (SD).

### Network visualization and statistical analysis

The networks in this study were displayed and analyzed by Gephi (v0.9.2, https://gephi.org/)^64^ and Cytoscape (v3.2.0, http://www.cytoscape.org/)^65^. The gene enrichment analysis was performed by ClueGO plug-in^32^ and OmicShare tools (http://www.omicshare.com/tools), and the statistical analysis and graphics were performed by R environment (v3.01, http://www.r-project.org/) and Python platform (v3.2, http://www.python.org/).

## Supplementary Information

Supplementary Information.

## Data Availability

The datasets generated during and/or analyzed during the current study are available from the corresponding author on reasonable request.
