# Supplementary material for: In silico identification of natural products from Traditional Chinese Medicine for cancer immunotherapy
Source: Sci Rep. 2021 Feb 8;11:3332. doi: 10.1038/s41598-021-82857-2 (PMC7870934; doi:10.1038/s41598-021-82857-2)
Supplement: Supplementary file 1 — Supplementary Information. [file 41598_2021_82857_MOESM1_ESM.zip › Supplementary material/Legends of Supplementary materialn.docx]

***In Silico* Identification of Natural Products from Traditional Chinese Medicine for Cancer Immunotherapy**

Chuipu Cai^1,2,+^, Qihui Wu^3,+^ , Honghai Hong^4,+^, Liying He^2^, Zhihong Liu^5^, Yong Gu^3^, Shijie Zhang^2^, Qi Wang^2^, Xiude Fan^6,*^, Jiansong Fang^2,*^

**Supplementary information**

**Figure Legends**

**Figure S1.** Chemical scaffolds clustering analysis of the 182 predicted positive natural products. **(A)** Statistics of the structures in the five cluster groups. **(B)** Structures of the five cluster centers**.** (.tif)

**Figure S2.** Circos plot exhibiting the 182 predicted natural products (adjusted-*P* < 0.01). Natural products are grouped by chemical scaffold clustering analysis. The predicted associations from different statistical network models are connected lines in various colors. Natural products validated by direct evidence and indirect evidence are highlighted in red and blue font, respectively. (.tif)

**Figure S3.** Systematic source analysis of the 182 predicted natural products. **(A)** Investigation of the species classification (class [the inner circle], order [the middle circle], and family [the outside circle]) of the 63 cancer immunotherapy related-TCMs according to the *Flora Reipublicae Popluaris Sinicae*. **(B)** Top 10 Chinese herbs (positive ingredients proportion > 10%) exhibiting highest number of predicted positive ingredients. (.tif)

**Figure S4.** Correlation analysis of the three sets of cancer immune response-related genes. (.tif)

**Table Legends**

**Table S1.** Literature mining results of the 66 herbs significantly correlated with cancer. (.xlsx)

**Table S2.** Compound-target interactions of the natural products isolated from the 66 cancer-related herbs. (.xlsx)

**Table S3.** Immune system process enrichment analysis results of the 41 cancer immune response-related genes. (.xlsx)

**Table S4.** Statistics of the predicted cancer immunotherapeutic natural products and relevant literature evidence. (.docx)

**Table S5.** Detailed information of the predicted cancer immunotherapy-related natural products and relevant literature evidence. (.xlsx)

**Table S6.** Combined Z-scores of the 49 natural products simultaneously predicted to be positive by all the five models. (.xlsx)

**Table S7.** Statistics analysis results of the positive ingredients amount and proportion in each herb. (.xlsx)

**Table S8.** Lists of the five sets of cancer immune response-related genes used in this study. (.xlsx)

**Figure S1**


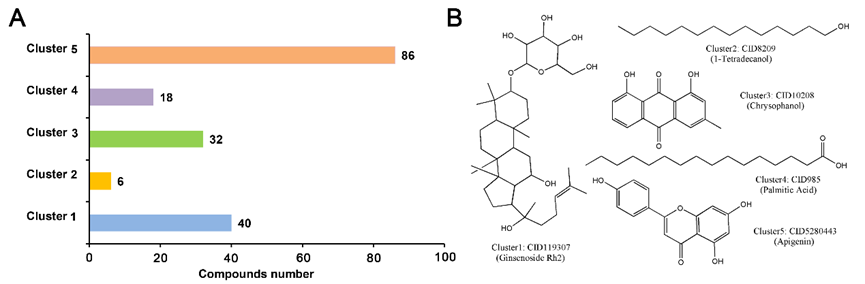


**Figure S2**

**
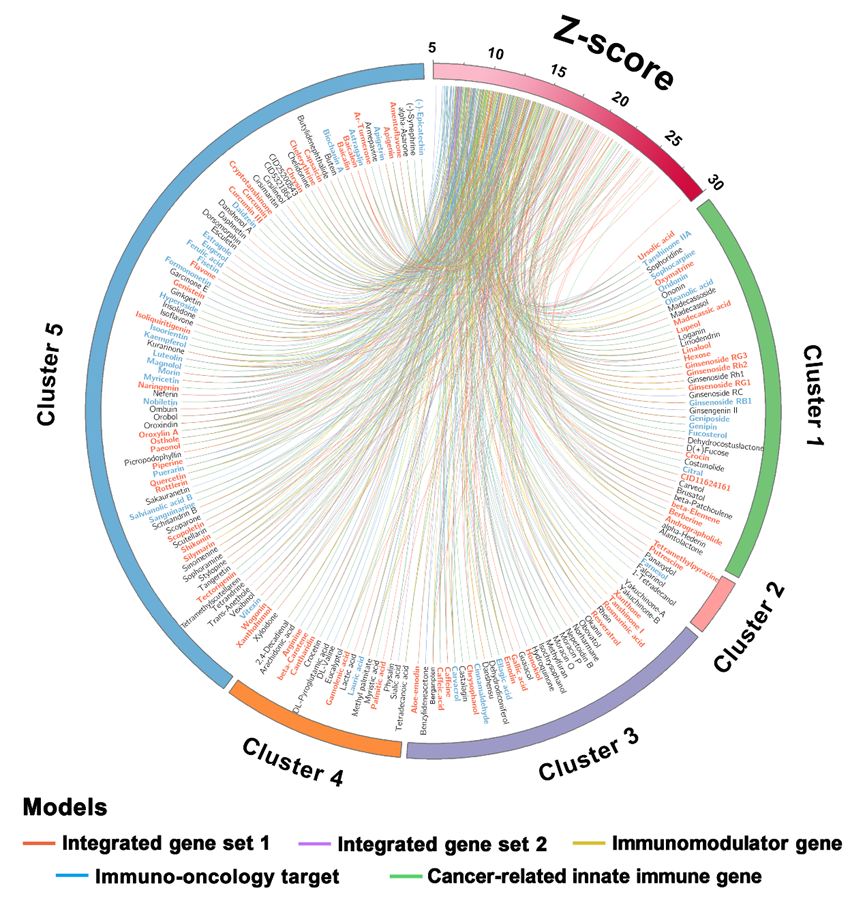
**

**Figure S3**

**
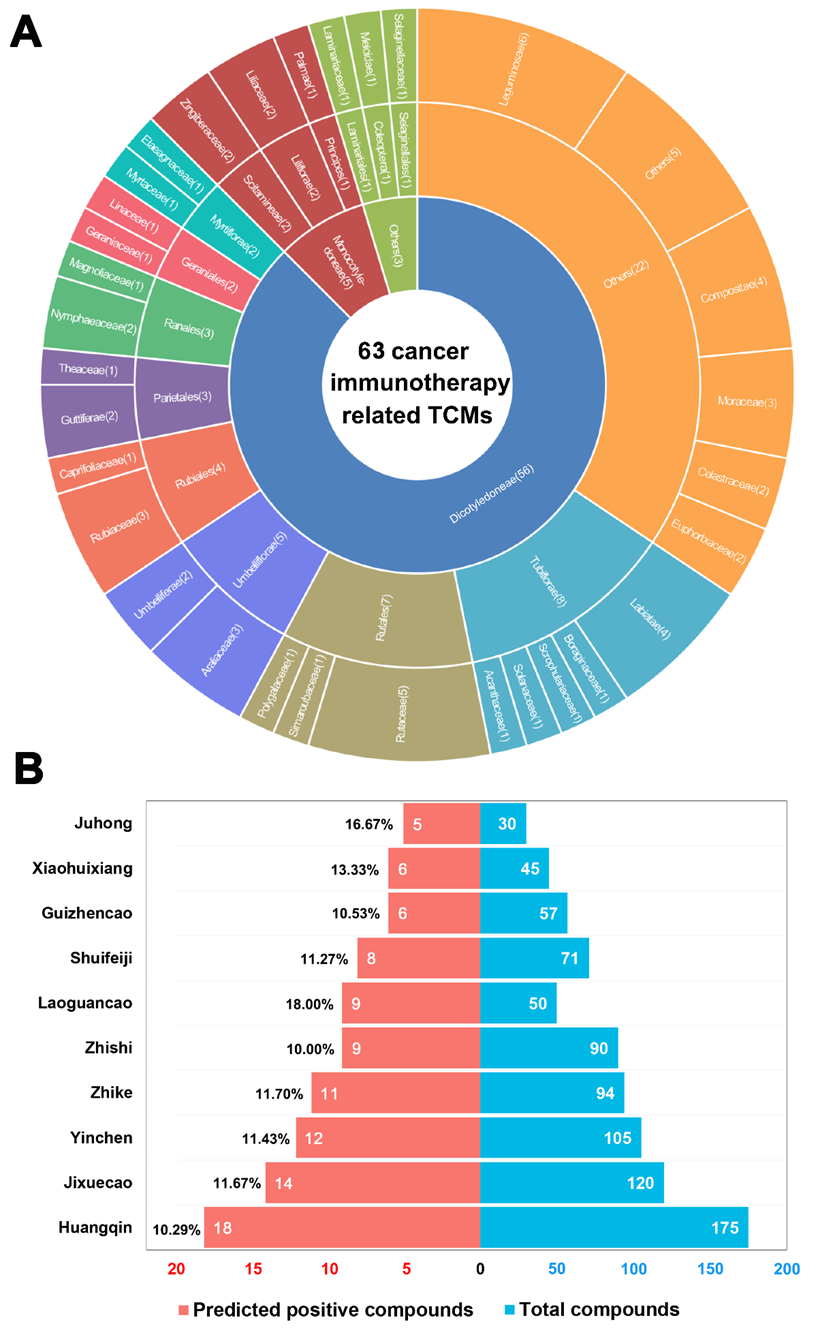
**

**Figure S4**

**
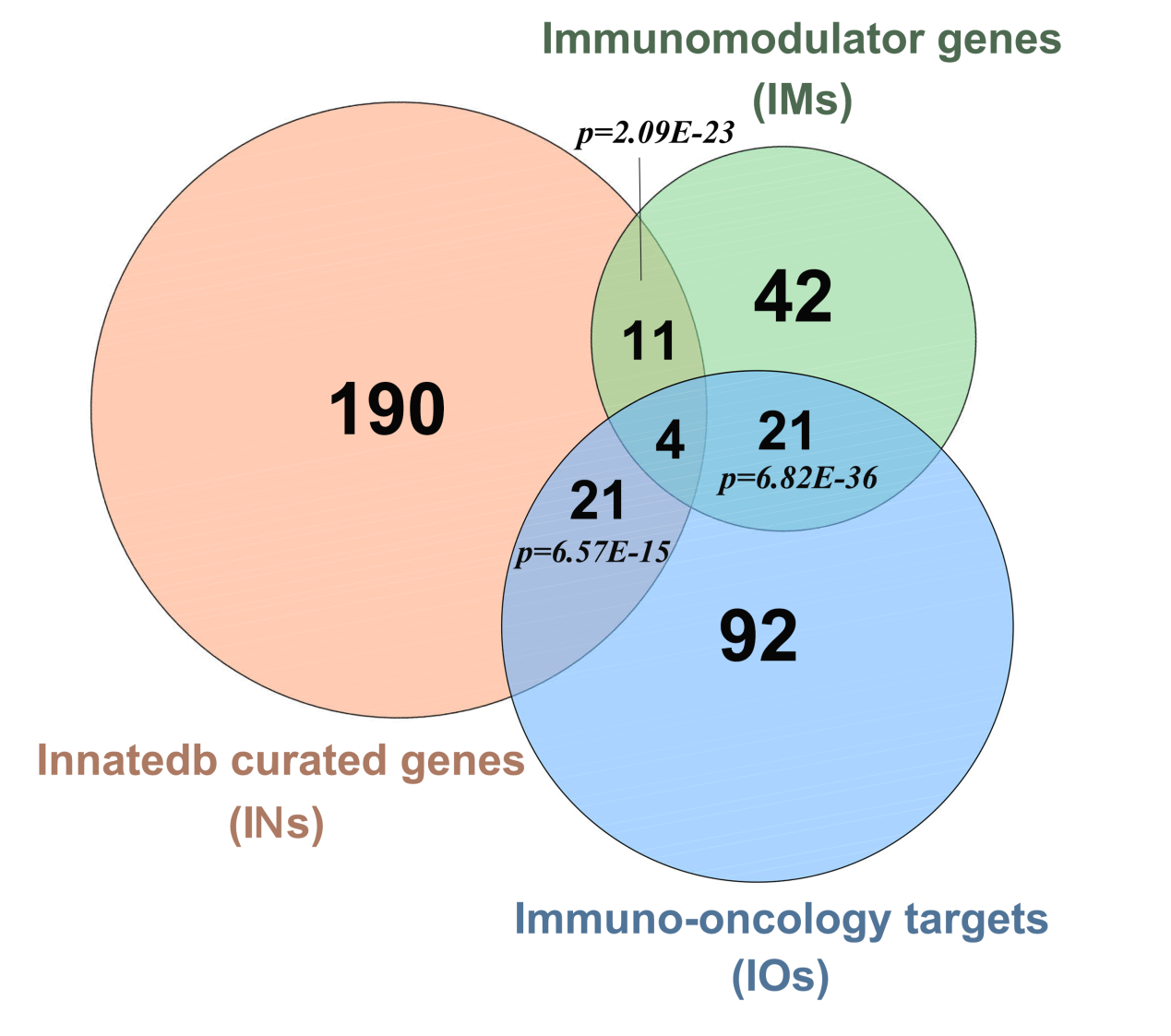
**
