# Supplementary material for: In silico identification of natural products from Traditional Chinese Medicine for cancer immunotherapy
Source: Sci Rep. 2021 Feb 8;11:3332. doi: 10.1038/s41598-021-82857-2 (PMC7870934; doi:10.1038/s41598-021-82857-2)
Supplement: Supplementary file 1 — Supplementary Information. [file 41598_2021_82857_MOESM1_ESM.zip › Supplementary material/Table S4.docx]

**Table S4.** Statistics of the predicted cancer immunotherapeutic natural products and relevant literature evidence.

| Model | Number of predicted compounds (*q*<0.01) | Number of predicted compounds validated by literature | | | Success rate |
| --- | --- | --- | --- | --- | --- |
|  |  | Direct evidence | Indirect evidence | Total |  |
| IMs | 71 | 25 | 14 | 39 | 54.93% |
| IOs | 100 | 34 | 22 | 56 | 56.00% |
| INs | 149 | 54 | 34 | 88 | 59.06% |
| IG-1 | 124 | 45 | 29 | 74 | 59.68% |
| IG-2 | 155 | 52 | 34 | 86 | 55.48% |

IMs: immunomodulator genes; IOs: immuno-oncology targets; INs: cancer-related innate immune genes; IG-1: integrated gene set 1; IG-2: integrated gene set 2.
