# Supplementary figures and images for: In silico identification of natural products from Traditional Chinese Medicine for cancer immunotherapy
Source: Sci Rep. 2021 Feb 8;11:3332. doi: 10.1038/s41598-021-82857-2 (PMC7870934; doi:10.1038/s41598-021-82857-2)

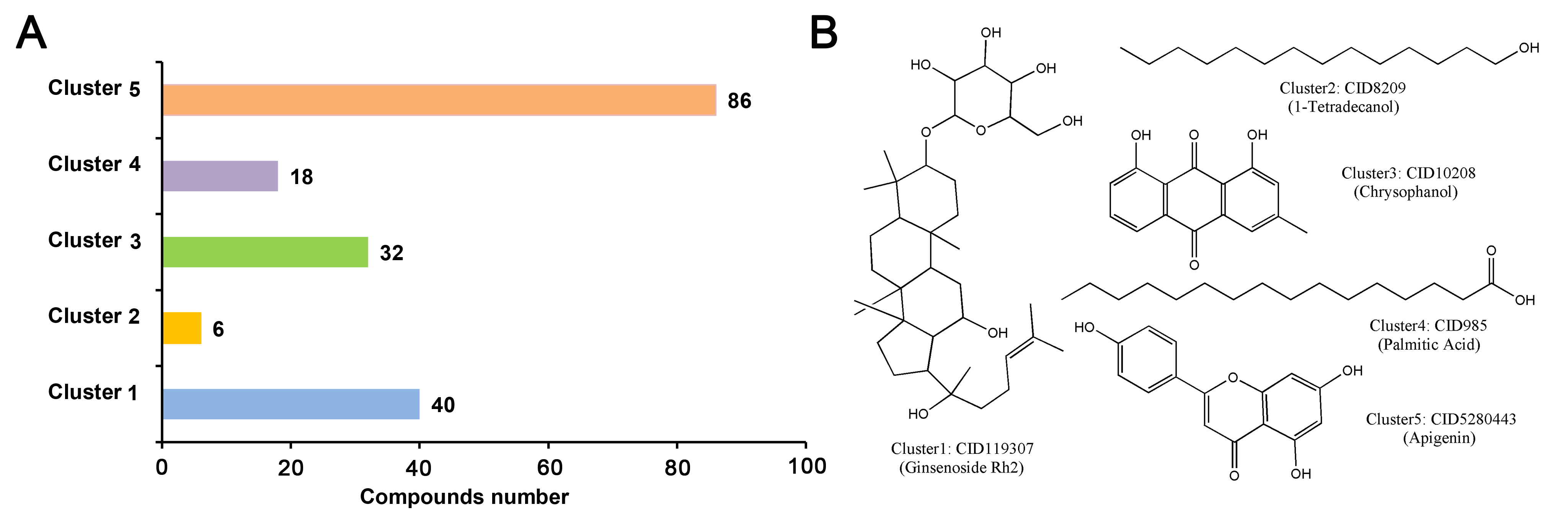

Supplement: Supplementary file 1 — Supplementary Information. [file 41598_2021_82857_MOESM1_ESM.zip › Supplementary material/Figure S1.tif]

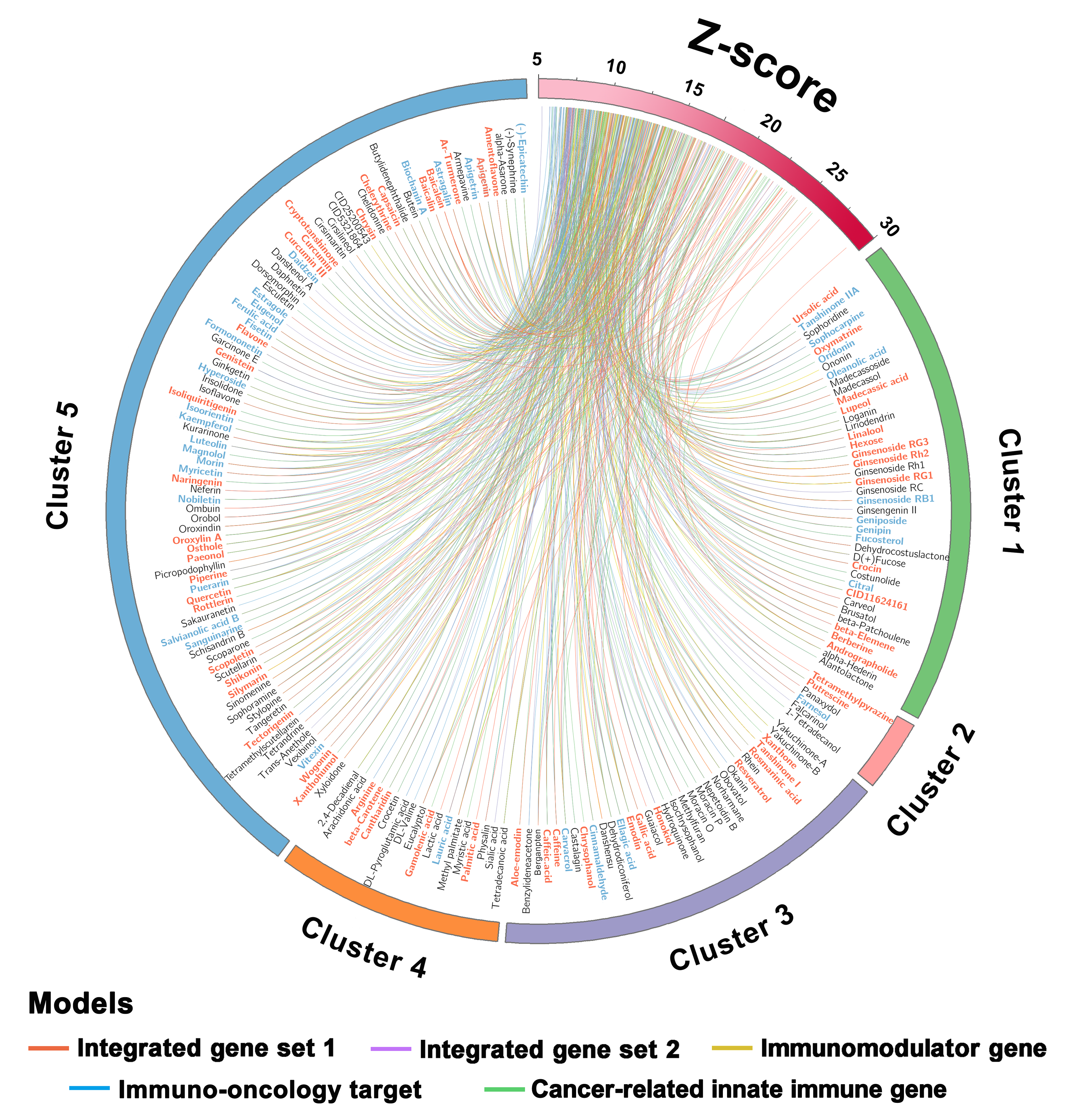

Supplement: Supplementary file 1 — Supplementary Information. [file 41598_2021_82857_MOESM1_ESM.zip › Supplementary material/Figure S2.tif]

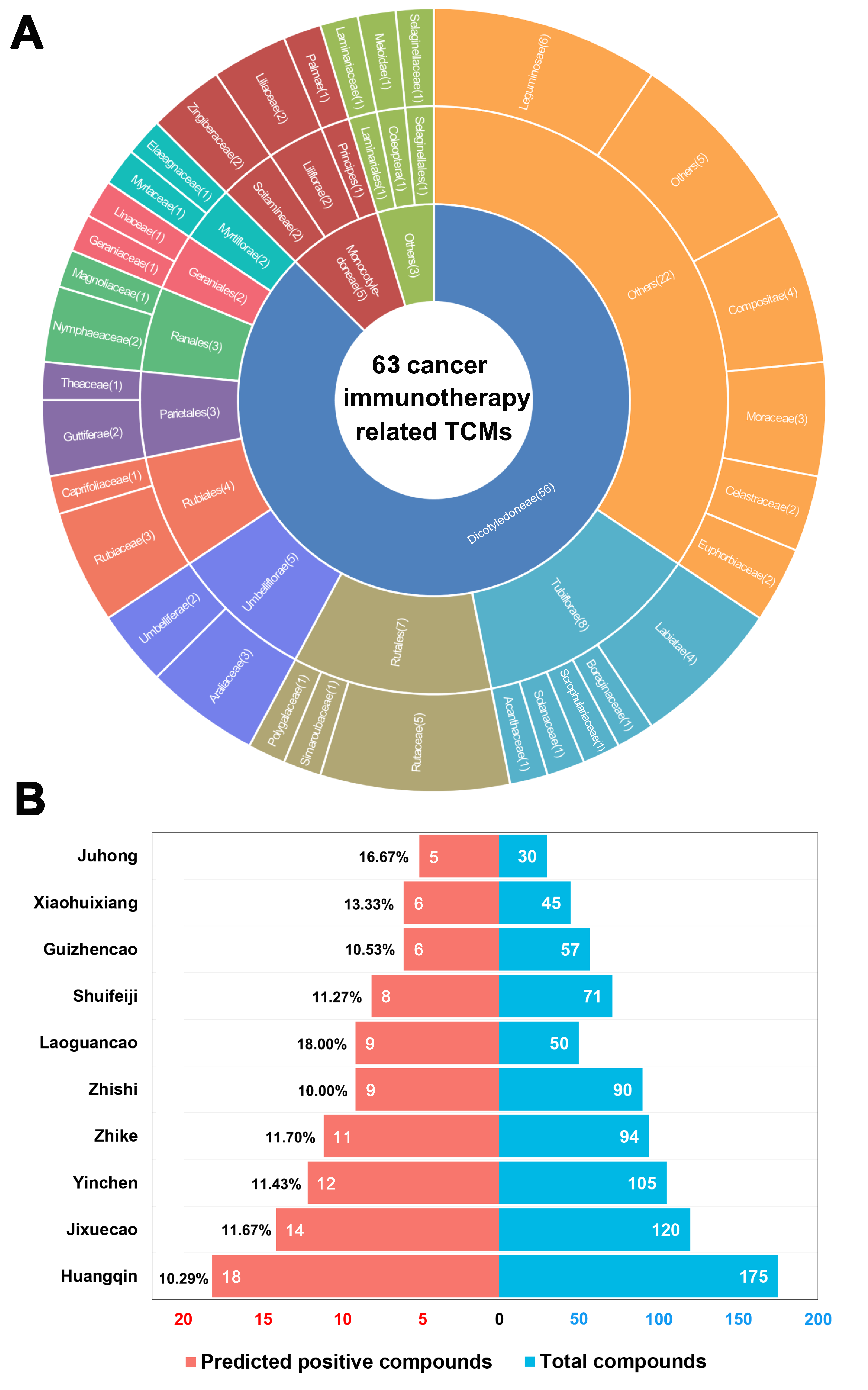

Supplement: Supplementary file 1 — Supplementary Information. [file 41598_2021_82857_MOESM1_ESM.zip › Supplementary material/Figure S3.tif]

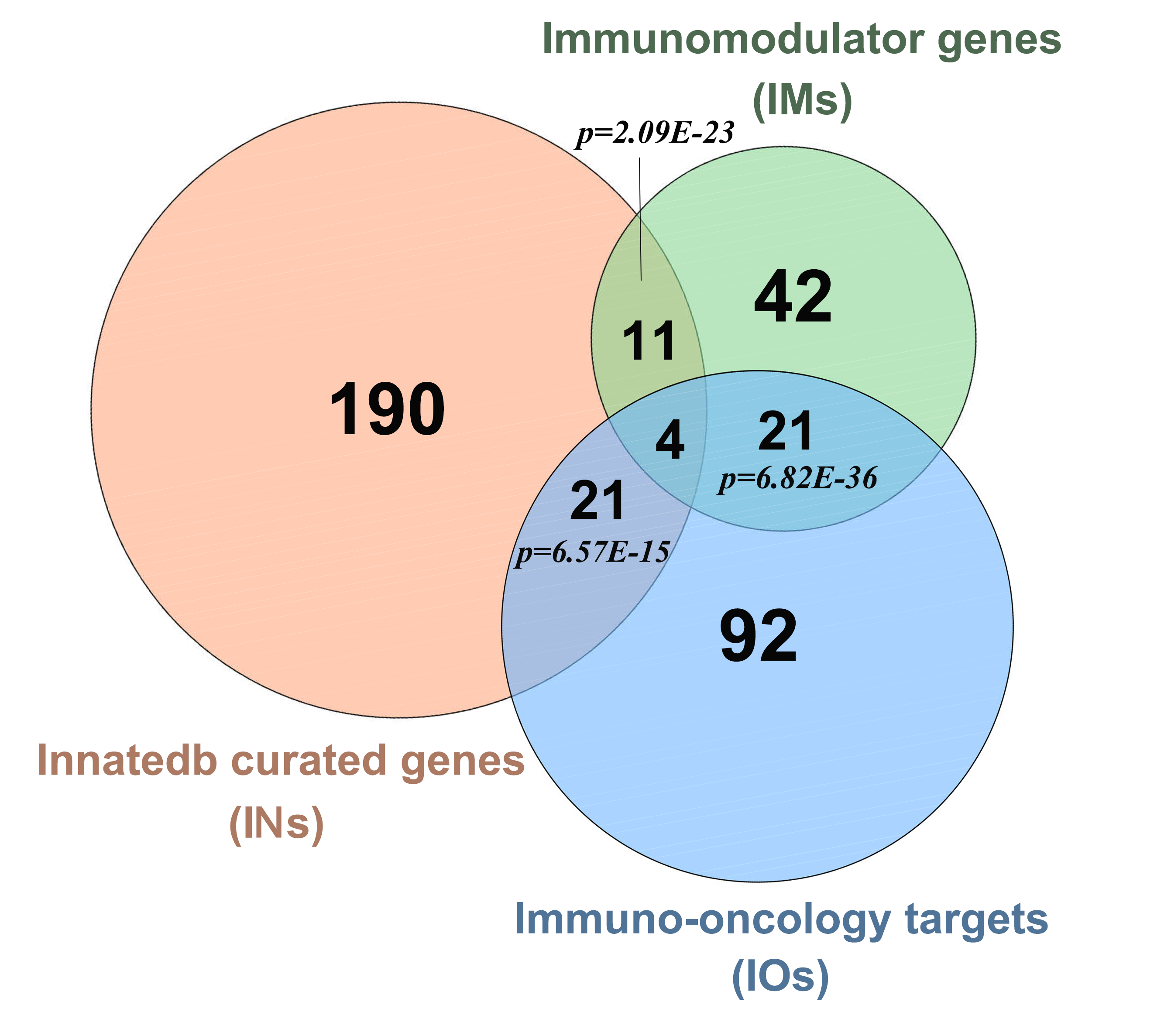

Supplement: Supplementary file 1 — Supplementary Information. [file 41598_2021_82857_MOESM1_ESM.zip › Supplementary material/Figure S4.tif]
